# A lasting impression

**DOI:** 10.7554/eLife.107620

**Published:** 2025-06-13

**Authors:** Rochelle Ackerley, Roger H Watkins

**Affiliations:** 1 Centre for Research in Psychology and Neuroscience (CRPN), Aix Marseille University - CNRS Marseille France

**Keywords:** somatosensation, skin mechanics, sensory afferent, touch, Human

## Abstract

Touch-sensitive neurons in the fingertips take previous physical contacts into account when relaying tactile information to the brain.

**Related research article** Saal HP, Birznieks I, Johansson RS. 2025. Memory at your fingertips: how viscoelasticity affects tactile neuron signaling. *eLife* **12**:RP89616. doi: 10.7554/eLife.89616.

Our hands are capable of performing a wide range of complex and delicate interactions with our environment. When we touch an object, specialized neurons in the skin – known as mechanoreceptors – provide real-time feedback on its size, shape, compliance, and texture. This tactile information is then rapidly relayed to the brain, allowing us to adjust and fine tune our grip on the object as required.

There are four main types of mechanoreceptors in the glabrous (non-hairy) skin covering the palm of our hands and fingers (Figure 1; see also Vallbo and Johansson, 1984). These neurons respond to physical changes to the skin with remarkable sensitivity and temporal precision, where even millisecond-level variations relay tactile information to the brain (Mackevicius et al., 2012).

**Figure 1.**
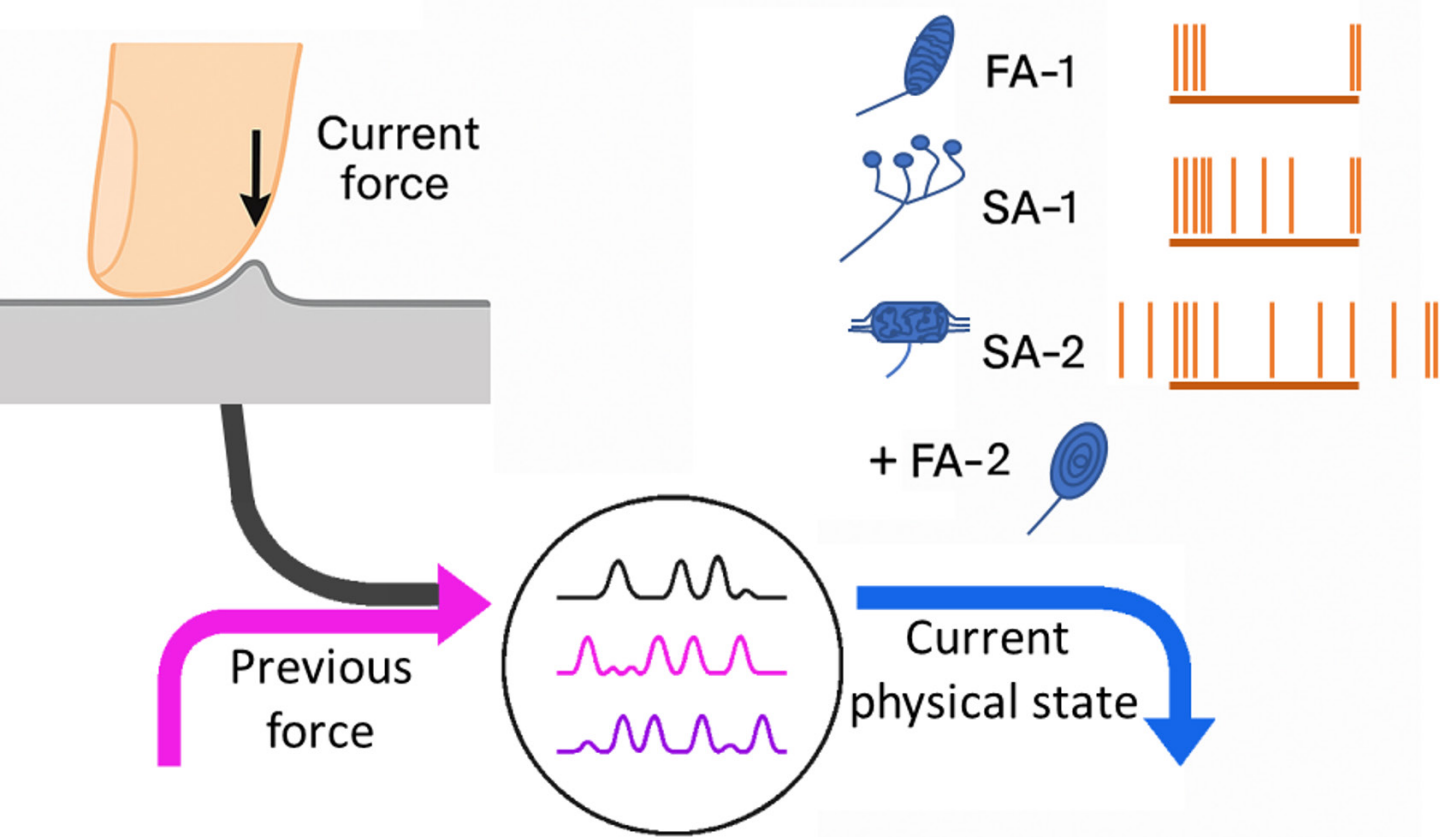
Previous touch shapes how current forces are represented by neurons in the fingertip. When one of our fingertips contacts a surface (top left), a series of forces are generated which alter the viscosity and elasticity of the skin. Saal et al. recorded how these changes influenced the firing pattern (vertical orange lines; top right) of touch-sensitive neurons called mechanoreceptors (dark blue shapes) that relay information from the skin to the brain. Data were mainly collected from three of the four types of mechanoreceptor found in the skin (FA-1, SA-1 and SA-2); the fourth type (FA-2) responded far less and was largely insensitive to different forces. Saal et al. found that the mechanoreceptors displayed different response patterns: some encoded the current force (black line; bottom), some encoded the previous force (pink), and some encoded both (purple). These neuronal signals are then combined across the population, influencing how information about the current physical state of the fingertip is conveyed to the brain (blue arrow).

Image Credit: Rochelle Ackerley.

In 2001, Ingvars Birznieks, Roland Johansson and others from Umeå University published a paper (Birznieks et al., 2001) which recorded the activity of individual mechanoreceptors in human glabrous skin using a technique called microneurography (Hagbarth and Vallbo, 1967). This vast dataset led to a number of important papers that have informed a lot of what is known about tactile signaling at the fingertip (Johansson and Birznieks, 2004; Saal et al., 2009). Now, in eLife, Hannes Saal (University of Sheffield), Birznieks (now at the University of New South Wales and Neuroscience Research Australia) and Johansson report how they built on this seminal work to show that forces exerted on to skin can leave a lasting imprint (Saal et al., 2025).

The team applied directional pressure at the fingertip of participants, and investigated how certain physical properties of the skin – viscosity and elasticity – were affected when the direction of the applied force was changed. These measurements were repeated, with the force shifting between five possible directions. Saal et al. found that the viscoelastic state of the skin at a given time depended not only on the direction of the current force, but also the direction of the force that preceded it.

The firing rate of individual mechanoreceptors was then recorded as the applied directional force rapidly changed with time. Again, the response was influenced by the previous force, suggesting that these neurons maintain a kind of ‘memory’ of the skin’s recent physical state. Interestingly, the mechanoreceptors varied in how they represented touch: some encoded the current force, some encoded the previous force, and some encoded both (Figure 1).

The study also discovered exciting new insights into a type of mechanoreceptor called SA-2 (short for slow-adapting type 2), which conveys information about skin stretch and is thought to contribute to the perception of pressure in the skin (Birznieks et al., 2009; Watkins et al., 2022). Saal et al. found that SA-2 neurons remained active between the different force directions, with this activity correlating with the physical state of the skin. This finding suggests that SA-2 mechanoreceptors provide ongoing feedback about the condition of the skin, which may help to correct for variability in the information conveyed by other types of mechanoreceptors.

Overall, the study by Saal et al. reveals the complex interplay between the physical properties of the skin and the neural encoding of touch. It suggests that the way you contact an object – such as the direction of the force applied – can influence the perception of future tactile interactions. Notably, a type of mechanoreceptor called FA-2 (short for fast-adapting type 2), which is good at sensing vibrations, was largely unaffected by the different forces, underscoring the specialized roles taken on by different types of mechanoreceptors and their distinct contributions to tactile signaling.

The findings also highlight the viscoelastic nature of the skin, demonstrated by how long it takes for skin to return to its baseline state after being touched. This physical property is dependent on various factors – such as the force of the touch, the hydration of the skin, and age – which need to be considered when interpreting the results of tactile experiments (Dione et al., 2023).

So far, the ‘skin memory’ effect described by Saal et al. has only been observed at the level of the peripheral nervous system. Further studies are needed to determine whether and how this phenomenon is represented by neurons in the brain. Understanding how physical properties of the skin affect mechanoreceptor signaling advances our knowledge of the neural mechanisms underlying touch. This could lead to exciting innovations that help improve the development of prosthetics, haptic interfaces, and robotic systems.
